# Coexistence of endometrial mesonephric-like adenocarcinoma and endometrioid carcinoma suggests a Müllerian duct lineage: a case report

**DOI:** 10.1186/s13000-019-0830-4

**Published:** 2019-06-07

**Authors:** Mitsutake Yano, Daisuke Shintani, Tomomi Katoh, Mei Hamada, Kozue Ito, Eito Kozawa, Kosei Hasegawa, Masanori Yasuda

**Affiliations:** 1 Department of Pathology, Saitama Medical University International Medical Center, 1397-1 Yamane, Hidaka-City, Saitama, 350-1298 Japan; 2 Department of Obstetrics and Gynecology, Oita University Faculty of Medicine, Oita, 879-5593 Japan; 3 Department of Gynecologic Oncology, Saitama Medical University International Medical Center, Saitama, 350-1298 Japan; 4 Department of Diagnostic Radiology, Saitama Medical University International Medical Center, Saitama, 350-1298 Japan

**Keywords:** Mesonephric-like adenocarcinoma, Endometrioid carcinoma, Endometrial cancer, Medroxyprogesterone acetate therapy, Müllerian duct

## Abstract

**Background:**

Endometrial mesonephric-like adenocarcinomas exhibit classical histologic features of mesonephric carcinoma; however, it remains unclear whether these tumors represent mesonephric (Wolffian) carcinoma or endometrioid (Müllerian) carcinomas that closely mimic mesonephric carcinoma.

**Case presentation:**

A 32-year-old Japanese primigravida presented with atypical vaginal bleeding. An endometrial biopsy suggested low-grade endometrioid carcinoma, and she was administered medroxyprogesterone acetate. Her tumor recurred 6 years later, and she underwent hysterectomy, salpingo-oophorectomy, and omentectomy, at which point she was diagnosed with mesonephric-like adenocarcinoma of the uterine endometrium. Retrospective pathological review of the initial biopsy confirmed coexisting low-grade endometrioid carcinoma and mesonephric-like adenocarcinoma of the uterine endometrium. On immunohistochemistry, the endometrioid carcinoma component was diffuse positive for estrogen and progesterone receptors but negative for thyroid transcription factor 1. However, the mesonephric-like adenocarcinoma component exhibited a mixture of estrogen receptor- and thyroid transcription factor 1-positive cells within the same glands.

**Conclusions:**

We encountered a patient with coexisting endometrial mesonephric-like adenocarcinoma and low-grade endometrioid carcinoma, which was treated using medroxyprogesterone acetate therapy, resulting in recurrence of mesonephric-like adenocarcinoma alone. These clinicopathological findings support the prevailing notions that mesonephric-like adenocarcinoma is a Müllerian adenocarcinoma exhibiting mesonephric differentiation.

## Background

Mesonephric carcinoma is a malignant gynecologic tumor most commonly arising in the cervix and is presumed to be derived from normal or hyperplastic mesonephric remnants [1]. Recently McFarland et al. [2] reported a series of 5 ovarian and 7 uterine endometrial neoplasms that were referred to as “mesonephric-like adenocarcinomas (MLAs)”; these tumors exhibited the classical histologic features of mesonephric carcinomas. MLAs are rare, representing approximately 1% of endometrial carcinomas, but appear to be aggressive in nature [3, 4]. Histologically, MLAs are characterized by a variety of morphologies including tubular, ductal, papillary, retiform, and solid. These histologic patterns can easily be mistaken for endometrioid carcinoma, clear cell carcinoma, serous carcinoma, carcinosarcoma, and a variety of other neoplasms [4, 5]. They also have a unique immunohistochemical profiles; they are usually positive for thyroid transcription factor 1 (TTF-1), GATA binding protein-3 (GATA-3), and CD10, and are negative for estrogen receptor (ER) and progesterone receptor (PgR) [2, 6]. MLAs are often confined to the endometrium without deep myometrial involvement, where mesonephric remnants theoretically exist [2]. MLAs harbor recurrent mutations in *KRAS* and *PIK3CA* but lack *PTEN* mutations, demonstrating biological overlap with both mesonephric and endometrioid carcinomas [7]. The pathogenesis of MLAs is unknown, and it remains debated whether they represent mesonephric (Wolffian) carcinomas arising in the endometrium/ovary or endometrioid (Müllerian) carcinomas that closely mimic mesonephric carcinomas. Herein, we report for the first time a case of a patient diagnosed with an endometrial MLA coexisting with a low-grade endometrioid carcinoma.

## Case presentation

### Clinical history

**Fig. 1:**
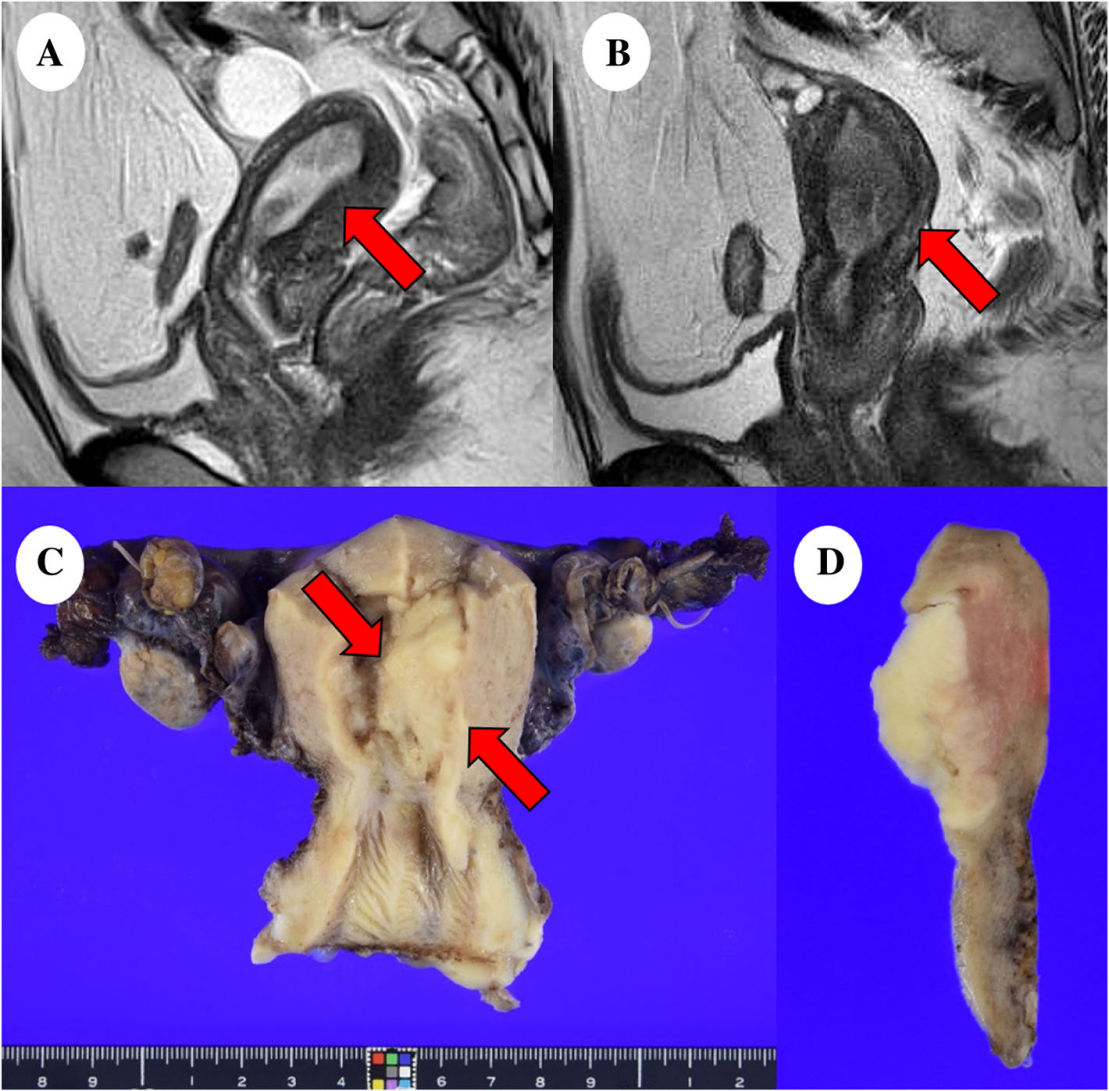
Magnetic resonance imaging and macroscopic analysis: (**a**) T2-weighted image of the initially diagnosed tumor (red arrow). (**b**) T2-weighted image of the recurred tumor (red arrow). (**c**) The endometrial mass (red arrows) was 40 × 23 mm-sized in the left wall of the uterine body and (**d**) had yellow-whitish cut surface

A 32-year-old Japanese primigravida visited another clinic because of atypical vaginal bleeding, wherein an endometrial mass was detected by transvaginal ultrasonography following which she was referred to our hospital for evaluation and treatment. Her body mass index was 33.8 kg/m^2^; magnetic resonance imaging (MRI) revealed the presence of a 25 mm mass at the uterine endometrium that was suspected to be endometrial cancer (Fig. 1a). No metastasis was detected using systemic computed tomography. Serum levels of the tumor markers carcinoembryonic antigen, cancer antigen 125, and carbohydrate antigen 19–9 were 1.2 ng/mL, 11.7 U/mL, and 7.9 U/mL, respectively. An endometrial biopsy suggested endometrioid carcinoma G1, which is categorized as low-grade endometrial carcinoma, and she received medroxyprogesterone acetate (MPA) therapy (600 mg/day) for 6 months. Afterwards, routine examination that included transvaginal ultrasonography, pelvic MRI, and endometrial cytology showed no evidence of the tumor. Six years after MPA therapy, an endometrial mass 24 mm in size was detected using MRI, indicating a recurrence (Fig. 1b). The patient underwent a total abdominal hysterectomy, bilateral salpingo-oophorectomy, and partial omentectomy. She has had no recurrence since the surgery (5 months).

### Pathological findings

**Fig. 2:**
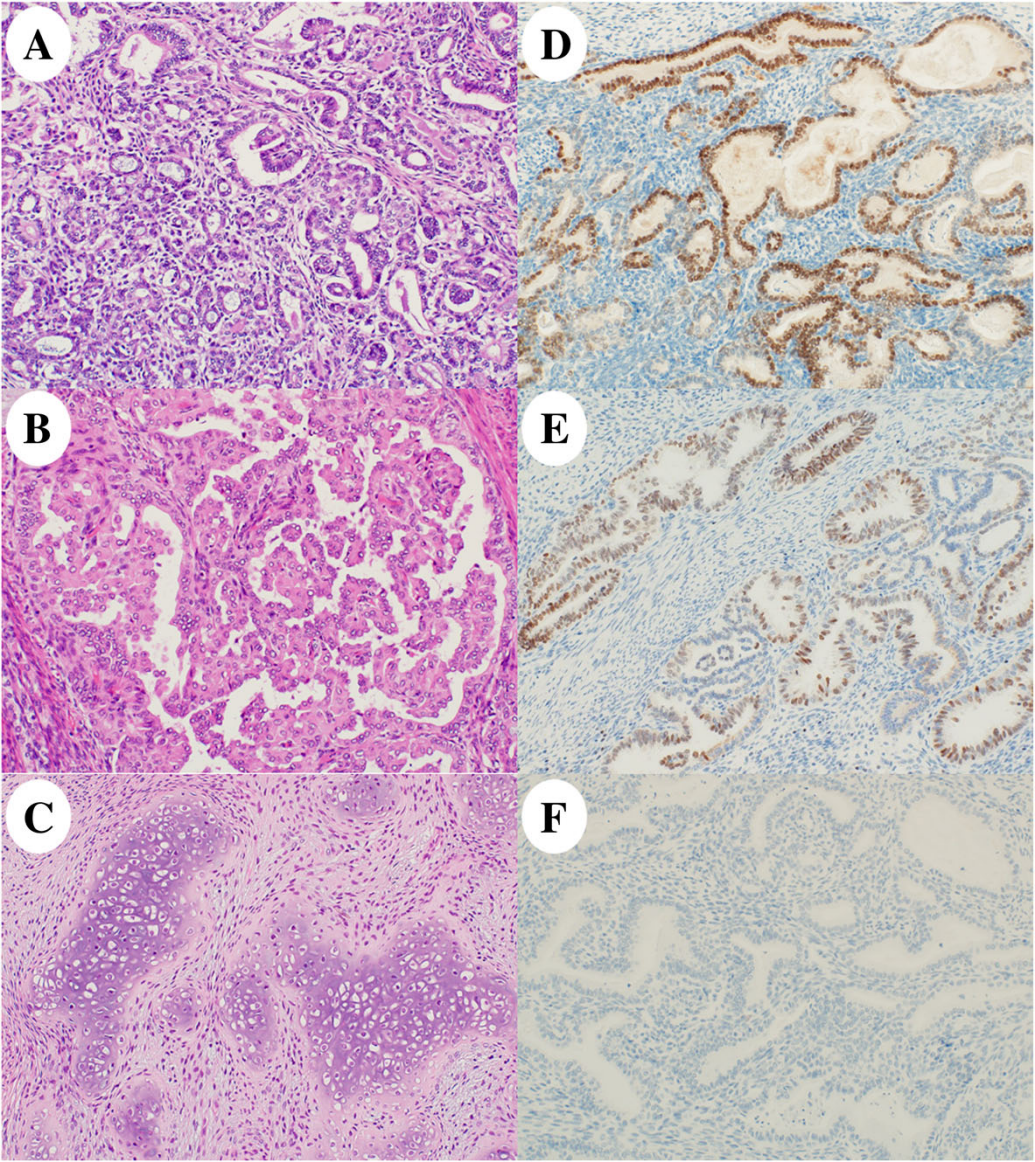
Histology and immunohistochemistry of the recurred tumor. The tumor had a variety of histologic patterns including (**a**, 20×) tubular and glandular, (**b**, 20×) papillary, and (**c**, 40×) spindled and solid with a heterologous element (cartilage without atypia). Immunohistochemical analysis showed positive staining for (**d**, 2 × 0) thyroid transcription factor 1 and (**e**, 20×) GATA-3; however, the tumor was negative for (**f**, 20×) estrogen receptor

The uterine body showed a tumor of the size 40 × 23 mm in the left wall with a yellow-whitish cut surface (Fig. 1c). The tumor was histologically found to be endometrial carcinoma with an unusual epithelial component exhibiting variable patterns such as tubular (30%), glandular (30%), papillary (5%), and solid (30%) structure, mimicking mesonephric carcinoma of the cervix (Fig. 2a, b). Additionally, the tumor had a heterologous element of cartilaginous cells (5%) with no atypia (Fig. 2c). The tumor was confined to the uterine body where the infiltration reached the inner half of the myometrium, but with no extension into the cervix, adnexae, and omentum (International Federation of Gynecology and Obstetrics stage IA [pT1a pNX cM0]). No associated mesonephric remnants or lesions were seen. No other neoplasias were present in the ovaries, fallopian tubes, cervix, or omentum. Immunohistochemical analysis of the surgical sample showed that the tumor cells were positive for TTF-1 (focal, strong), GATA-3 (focal, strong), CD10 (focal, strong), p53 (focal, weak), CA125 (focal, strong), CK7 (diffuse, strong), and p16 (focal, strong). Moreover, they were negative for ER, PgR, calretinin, hepatocyte nuclear factor-1-β, napsin A, androgen receptor, and Wilms’ tumor 1 (WT-1) protein (Fig. 2d–f) (Table 1). On molecular testing, *KRAS* mutation (c.35G > C, p.G12A; Gly12Ala) was detected in this endometrial cancer.

**Table 1:** List of antibodies

Antigen	Clone	Dilution	Manufacturer
Estrogen receptor	SP1	1:1	Ventana, AZ, USA
Progesterone receptor	1E2	1:1	Ventana, AZ, USA
TTF-1	8G7G3/1	1:50	Dako, Kyoto, Japan
GATA-3	L50–823	1:1	Ventana, AZ, USA
CD10	56C6	1:40	Leica Biosystems, Wetzlar, Germany
p53	DO-7	1:50	Dako, Kyoto, Japan
CA125	M11	1:50	Dako, Kyoto, Japan
CK7	OV-TL 12/30	1:100	Dako, Kyoto, Japan
p16 (INK4)	G175–405	1:10	Becton Dickinson, NJ, USA
Calretinin	5A5	1:100	Leica Biosystems, Wetzlar, Germany
HNF-1β	Poly	1:400	SIGMA, Kanagawa, Japan
Napsin A	TMU-Ad02	1:50	IML, Gunma, Japan
Androgen receptor	AR441	1:50	Dako, Kyoto, Japan
WT-1	6F-H2	1:50	Dako, Kyoto, Japan

*TTF-1* thyroid transcription factor 1, *GATA-3* GATA binding protein 3, *CA125* cancer antigen 125; HNF-1β, hepatocyte nuclear factor-1β

**Fig. 3:**
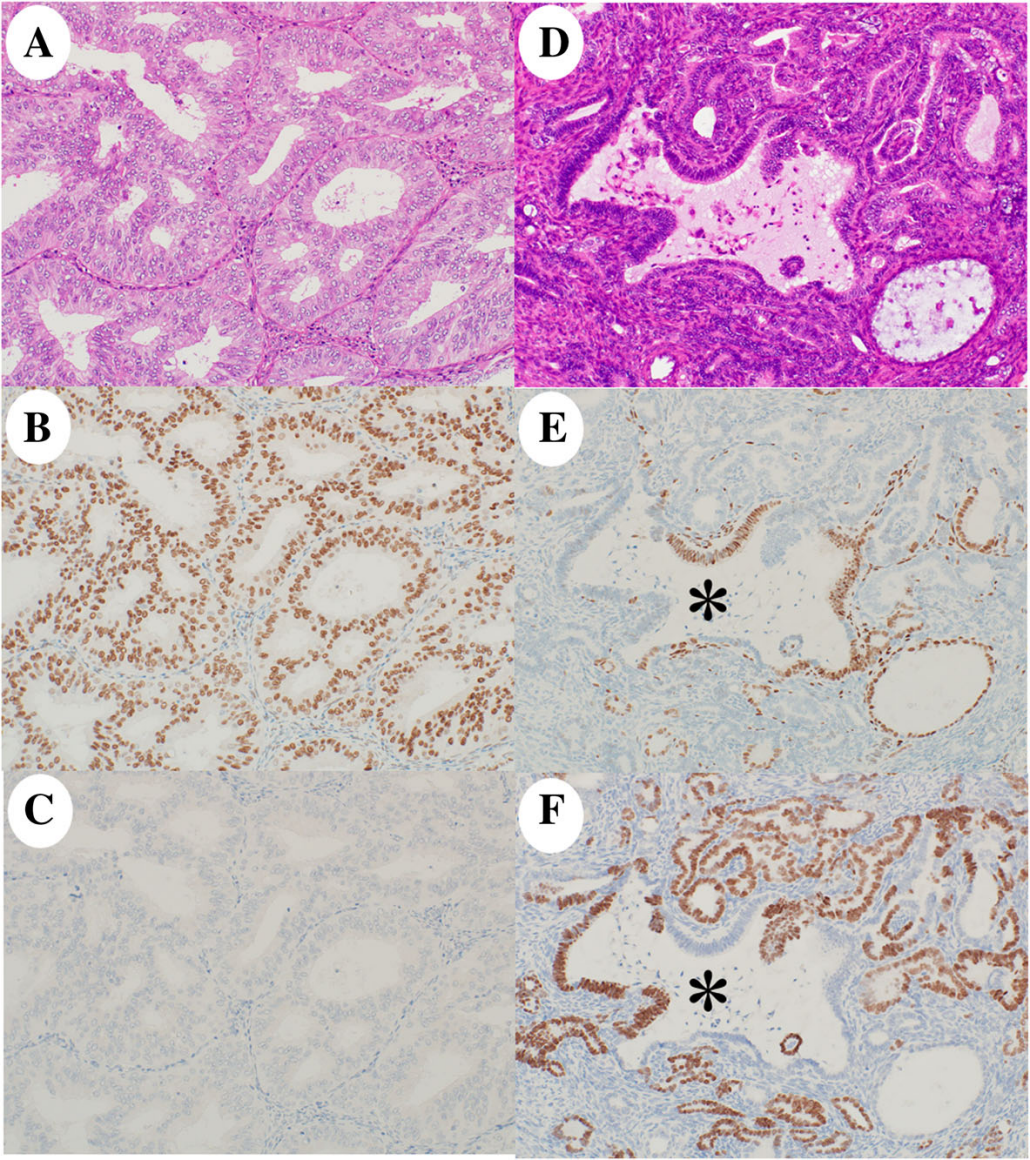
Histology and immunohistochemistry of the initially diagnosed tumor. The tumor had (**a**, 20×) a low-grade endometrioid carcinoma component and (**d**, 20×) a mesonephric-like adenocarcinoma component. Immunohistochemically, the endometrioid carcinoma was diffuse positive for (**b**, 20×) estrogen receptor (ER) and negative for (**c**, 20×) thyroid transcription factor 1 (TTF-1). The mesonephric-like adenocarcinoma showed a transition pattern with a mixture of cells positive for (**e**, 20×) ER- (a Müllerian duct marker) and (**f**, 20×) TTF-1- (a Wolffian duct marker) within the same glands (asterisk)

The initial endometrial biopsy showed the following: the tumor was found to have a coexistence of usual and unusual carcinomas in which the former was a low-grade endometrial carcinoma, i.e., endometrioid carcinoma G1 (70%), and the latter was composed of the same components as the surgical sample (30%) (Fig. 3a). On immunohistochemical analysis of the initial biopsy (Table 1), the endometrioid carcinoma component was diffuse positive for ER and PgR, but negative for TTF-1 and GATA-3 (Fig. 3b, c). The unusual epithelial components showed a transitioning pattern with a mixture of ER- and TTF-1-positive cells within the same glands, representing Müllerian and Wolffian duct markers, respectively (Fig. 3d–f). On molecular testing, same *KRAS* mutation (c.35G > C, p.G12A; Gly12Ala) was detected in the initial biopsy.

Based on these findings, a coexistence of endometrioid carcinoma G1 and MLA in the uterine body was diagnosed at the biopsy. After MPA therapy, tumor composed of only the component of MLA was found as recurrence.

## Discussion and conclusions

Endometrial MLA occurs in patients of all ages; the results of some studies show that approximately 40 cases with MLA were been reported in women of ages between 31 and 91 years [2, 3, 6]. Furthermore, it can mimic various other endometrial cancers. In women of reproductive age, low-grade endometrial carcinoma, represented by endometrioid carcinoma G1/2, is treated with MPA to preserve fertility [8]. The MLA in our patient was coincidentally treated with MPA therapy because of misdiagnostic consideration of endometrioid carcinoma G1; to our best knowledge, there have been no previous reports with MLA which was treated with MPA. This therapy produced a good response for the endometrioid carcinoma, resulting in a complete disappearance. However, MPA therapy did not suppress the recurrence of MLA. The discrepancy in the effect of therapy between the endometrioid carcinoma G1 and MLA is considered to be attributed to overexpression of hormone receptors (ER and PgR) in the former component and weak expression of them in the latter component. Poor MPA efficacy is usually associated with weak or no expression of ER and PgR [8, 9]; hence, MPA therapy appears to be ineffective against MLA. Therefore, MLA needs to be distinguished from endometrioid carcinoma in terms of selection of appropriate treatments.

MLA is often misdiagnosed as other malignant neoplasms, due to its unfamiliar nature. Microscopically, MLA is consistently heterogeneous in its architecture (tubular, papillary, sieve-like, ductal, solid) with cuboidal to columnar epithelium and attenuated segments [5]. Occasional small ducts or tubules can contain intraluminal eosinophilic sections. The cells tend to have frequently scant eosinophilic cytoplasm [5]. When such morphology is observed, additional studies are required in the routine clinical setting. The following panel-based immunohistochemistry results are important for differential diagnosis between endometrioid carcinoma and endometrial MLA: positive for TTF-1, GATA-3, and CD10; negative/partially positive for ER and PgR; and showing wild type p53 expression. Endometrial MLA is usually positive for TTF-1 but is more frequently ER positive and/or GATA-3 negative than cervical mesonephric carcinoma [4, 6]. The present patient also is characterized as focally containing a heterologous component of cartilaginous tissue; while cervical mesonephric carcinoma with heterologous elements such as cartilage and/or skeletal muscle were previously reported, none with endometrial MLA containing heterologous elements has been described to date [6, 10]. The present case provides evidence that even with heterologous elements it is not necessarily carcinosarcoma. We believe that carcinosarcoma could be ruled out based on the mild atypia of the cartilaginous tissue.

It remains to be clarified whether MLA represents essentially mesonephric (Wolffian) carcinoma or mesonephric carcinoma presenting like endometrioid (Müllerian) carcinoma. The present case with MLA was speculated to have a closely Müllerian lineage based on the two evidences as follows: the coexistence with endometrioid carcinoma of Müllerian duct origin and the immunohistochemical admixture/transition between ER- and TTF-1-positive cells within the same glands. In a series of patients with uterine neoplasms, McFarland et al. [2] found that MLAs predominantly involved the endometrium from which they appeared to arise, and showed subsequent invasion into the myometrium; none involved the myometrium without endometrial involvement. Moreover, 3 of their 5 patients with ovarian MLA had foci of endometriosis admixed with or adjacent to the carcinoma. Chapel et al. [11] also reported an ovarian carcinoma with combined low-grade serous and mesonephric morphologies that were indicative of a Müllerian origin. Taken together, these MLA features all suggest a Müllerian neoplasm rather than a true Wolffian/mesonephric tumor.

In conclusion, we encountered a patient with coexisting endometrial MLA and low-grade endometrioid carcinoma, which was treated using MPA therapy, resulting in recurrence of MLA alone. These clinicopathological findings support the prevailing notions that MLA is a Müllerian adenocarcinoma exhibiting mesonephric differentiation.

## Abbreviations

ER: Estrogen receptor
GATA-3: GATA binding protein-3
MLA: Mesonephric-like adenocarcinoma
MPA: Medroxyprogesterone acetate
MRI: Magnetic resonance imaging
PR: Progesterone receptor
TTF-1: Thyroid transcription factor 1
